# Paraneoplastic Neurological Syndromes: Severe Neurological Symptoms Resulting from Relatively Benign or Occult Tumours—Two Case Reports

**DOI:** 10.1155/2013/458378

**Published:** 2013-04-08

**Authors:** M. Ghadiri-Sani, Mueez Waqar, Dave Smith, Mark Doran

**Affiliations:** ^1^Neurology Registrar, Walton Centre for Neurology and Neurosurgery, Lower Lane, Fazakerley, L9 7LJ Liverpool, UK; ^2^School of Medicine, University of Liverpool, L69 3GE Liverpool, UK; ^3^Consultant Neurologist, Walton Centre for Neurology and Neurosurgery, Lower Lane, Fazakerley, L9 7LJ Liverpool, UK

## Abstract

*Introduction*. Paraneoplastic syndromes represent rare symptom complexes resulting from the ability of tumour cells to disrupt the homeostatic processes of various bodily systems. Here we present two cases to demonstrate how such tumours may evade detection even after extensive investigation and how even relatively benign tumours can produce severe neurological symptoms. *Case 1*. A 69-year-old female was admitted with a subacute onset of dysarthria, ataxia, and cerebellar signs. Workup revealed a relatively benign Non-Hodgkin's Lymphoma. *Case 2*. A 64-year-old female was admitted with acute leg weakness, which progressed to quadriplegia and was eventually fatal over the ensuing months. Her Ca-125 was elevated, though three different CT views of her pelvis and surgical exploration failed to demonstrate any malignancy. *Discussion*. These cases highlight how even relatively benign or very small tumours may result in severe neurological symptoms. Suspecting and investigating paraneoplastic syndromes (PNSs) are crucial as up to 80% of patients present with PNS before there is any other indication of malignancy. A PET scan and regular surveillance may reveal occult malignancies better than CT or MRI. Neuromodulatory therapies and treatment of the underlying malignancy remain the best management options in these patients.

## 1. Introduction

The term paraneoplastic was first used by Guichard and Vignon in 1949 to describe a patient presenting with multiple cranial nerve and radicular neuropathies [1]. A distinction must be made between these disorders and the clinical features produced by metastasis, which may be due to biomechanical disruption and other nonneoplastic complications of cancer, such as coagulopathy and nutritional imbalance [2]. Paraneoplastic neurological syndromes (PNSs) usually involve some degree of molecular mimicry, in which tumour cells display antigenic sequences homologous to those found in the central nervous system [2]; the ensuing antibody and cell-mediated responses result in the collateral damage of neuronal structures [2]. Although their collective incidence is under 1%, PNS may occur more frequently in certain malignancies, including lymphoma (10%) and small cell lung cancer (5%) [3]. Prognosis too depends on tumour type, though it is noteworthy that, in almost 80% of cases, the PNS represents the initial presentation of an as yet undiagnosed tumour [3]. Here we present two cases to demonstrate how such tumours may evade detection even after extensive investigation and how even relatively benign tumours can produce severe neurological symptoms.

## 2. Case 1

### 2.1. Presentation

A 69-year-old Caucasian female with no past medical history of note was admitted with a two-month history of progressive ataxia, impaired balance, and slurring of speech, associated with emotional liability. There was no family history, and she denied any systemic symptoms such as a headache. She was a nonsmoker, who lived with her partner, drinking alcohol only on occasion. On examination she displayed emotional liability and cerebellar signs including high pitched scanning speech, down beat vertical nystagmus, dysmetria, past pointing, dysdiadokinesis, truncal ataxia, and marked titubation of neck and trunk which impeded her gait. Visual acuity was 6/18 in the right eye and 6/9 in the left eye with intact visual fields and bilaterally pale discs. Examination of the upper and lower limbs revealed increased tone with brisk reflexes throughout and extensor plantars. There was no evidence of motor signs, bowel or bladder dysfunction. Systemic examination including breast examination was normal.

### 2.2. Investigations

The patient had several blood tests, of which only B12 and ESR appeared to be abnormal at 142 ng/L (normal > 150 ng/L) and 80 mm, respectively. Other parameters, including immunoglobulins, protein electrophoresis, autoimmune and vasculitic screens, antineuronal antibodies, and tumour markers, were all negative. Her MRI revealed hyperintensities in the medial temporal lobes consistent with limbic encephalitis but was otherwise unremarkable, revealing no evidence of cerebellar or brainstem pathology or atrophy. She then went on to have a CSF examination; this revealed an IgG of 49, positive oligoclonal bands and weakly positive protein 14 : 3 : 3, with other parameters being within the normal range. 

### 2.3. Treatment and Progression

The patient was treated empirically for immune mediated subacute combined cerebellar degeneration, with high dose steroids with very little improvement. This prompted a 5-day course of plasma exchange, though the patient's condition remained unstable, and she underwent a further 3 courses, all with minimal improvement. A more extensive search for a possible tumour was then conducted. Although transvaginal ultrasound was normal, mammography did reveal a noticeably enlarged lymph node in the right axilla and a poorly defined lesion at 11 o'clock to the nipple. FNA of the former determined it to be benign, and as for the latter, histology was consistent with a papillary change, with subsequent core biopsy being unremarkable. 

Further radiological investigations were conducted, including a CT of her chest, abdomen, and pelvis, revealing a single intra-abdominal lymph node. She underwent a laparotomy and lymph node biopsy, which revealed in the discovery a grade 3A follicular lymphoma. She received immunotherapy, steroids followed by azathioprine but no specific chemotherapy for the lymphoma. Her neurological status remains essentially unchanged. 

## 3. Case 2

### 3.1. Presentation

A 64-year-old Caucasian female presented with sudden onset of unilateral leg weakness, where she experienced great difficulty in hip flexion. She had previously been well but had recently complained of excessive fatigue and episodes of abdominal pain. Examination revealed a vasculitic rash covering extensor surfaces of her knees; proximal left leg weakness was also demonstrated. The patient was admitted with a provisional diagnosis of dermatomyositis, her blood results revealing a grossly elevated CK of 15,000 u/L (normal 25–170 u/L), and she was treated empirically with high dose steroids. Unfortunately, this failed to control her weakness, which gradually progressed over 8 weeks to involve both lower limbs. She additionally developed a bulbar palsy and weakness of neck flexion, prompting her transfer to ITU. 

On admission to ITU, patient was moribund, with bulbar dysarthria, nasal speech, and a nonpruritic, vasculitic rash covering the extensor surfaces of her arms and knees. There was no evidence of Gottron's sign or of a heliotropic rash. Examination of her limbs elicited significant weakness, more severe proximally. 

### 3.2. Investigations

**Figure 1:**
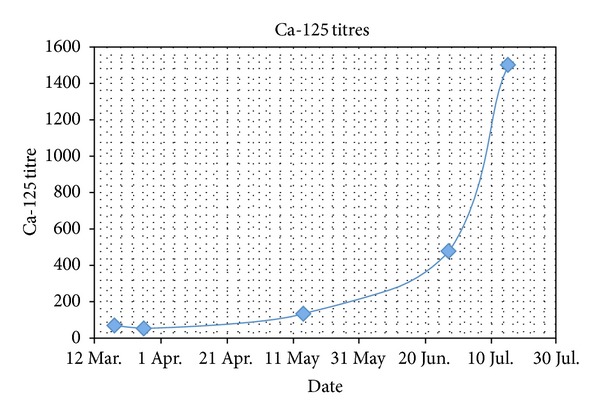
Ca-125 levels.

An EMG revealed a myodestructive pattern, with reduced amplitudes on NCS. A muscle biopsy was also performed, revealing atrophic sclerotic muscle, which was histologically consistent with dermatomyositis. Throughout her admission, the patient had several investigations, including routine bloods, glucose, TFTs, B12 and folate, autoantibodies, iron studies, and tumor markers; the only abnormality appeared to be an elevated Ca-125 (see Figure 1). CT of her pelvis failed to reveal any malignancy on three different occasions.

### 3.3. Treatment and Progression

Whilst in ITU, the patient additionally developed complex partial seizures originating from the left occipitotemporal region, though she had a normal CT head and only evidence of small vessel disease on MRI. A maintenance regimen of propofol and phenytoin seemed to control her seizures. She then developed what appeared to be an acute abdomen, for which a laparotomy was arranged; despite exploration revealing inflammatory changes involving her right adnexa, no malignancy was identified, and she was transferred back to ITU and eventually to a ward. 

The patient's weakness was refractory to immunoglobulins and methotrexate, though it showed a dramatic response to rituximab, and plans were made for her discharge, during which time she developed a DVT with a concurrent PE, despite pharmacological and mechanical measures. Therefore a PET scan was arranged upon her discharge. Before this could be done, the patient presented with disseminated ovarian carcinoma, which was ultimately fatal.

## 4. Discussion

Suspecting and investigating paraneoplastic syndromes (PNSs) are crucial as up to 80% of patients present with PNS before there is any other indication of malignancy [5]. The majority of paraneoplastic disorders are thought to be immune mediated, and, thus far, several antibodies with associate syndromes have been discovered affecting various parts of the nervous system (see Table 1). 

The cerebellum is commonly involved in paraneoplastic syndromes. The extent of the involvement, clinical course, and the prognosis depends on the associated antibody and the underlying tumour [6]. The most commonly associated tumours are gynaecological and breast cancer (anti-Yo and anti-Ri), lung cancer (anti-Hu), and Hodgkin's lymphoma (anti-Tr and anti-mGluR1) [6]. Majority of these patients present with subacute cerebellar ataxia (all antibodies), nystagmus (anti-Ri), and noncerebellar features (Anti-Hu) [6]. Treating the underlying malignancy does have some benefit in improving neurological features and survival in some cases, especially those with anti-Ri antibodies and particularly worse in anti-Yo antibodies. In our patient, cerebellar signs and limbic encephalitis were the presenting features of Non-Hodgkin's Lymphoma. Due to a high clinical suspicion we were able to persuade the general surgical team to carry out a biopsy of the solitary node identified on CT CAP to diagnose the patient, even when her antineuronal antibody screen was negative. 

Dermatomyositis is an autoimmune myopathy which typically features characteristic skin changes in association with subacute proximal muscle weakness, elevated muscle enzymes, and histological changes consistent with inflammation or necrosis [7]. Skin changes most specific for dermatomyositis include a purplish discolouration distributed periorbitally (heliotrope rash) and an erythematous rash distributed over the extensor surfaces of the interphalangeal joints (Gottron's papules) [7]. Almost a third of patients may be found to have an underlying malignancy, most commonly of the ovaries, lung, and pancreas [8]. Management strategies are largely immunosuppressive, with steroids and steroid-sparing agents commonly being employed. Importantly, an underlying tumour should be suspected in cases which are difficult to treat, to prevent potentiation of carcinogenesis by immunosuppression, which may have occurred with the use of rituximab in the 2nd case [9]. This is a subtle point: immunosuppressive therapy may result in progression of the tumour though mechanism whereby the patient's immunity is compromised, including the “protective” role of the paraneoplastic antibodies against the tumour cells.

**Figure 2:**
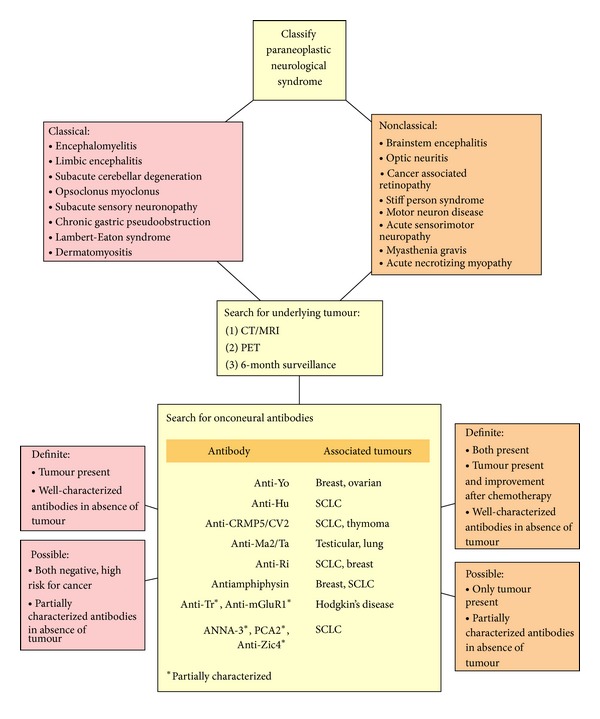
Diagnostic guidelines for paraneoplastic syndromes (adapted from source) [4]. Not all nonclassical syndromes have been listed, and, for a complete list, please see source.

Recommended diagnostic guidelines exist to ascertain the likelihood of a neurological presentation representing one of the paraneoplastic syndromes (see Figure 2) [4]. The preceding cases fulfill the criteria for a “definite” diagnosis, given the presence, in each, of a classical neurological syndrome accompanied by a diagnosis of cancer in under 5 years [4]. Given the currently proposed immunological model of PNS, it may seem paradoxical that both patients had undetectable levels of onconeural antibodies. In fact, these antibodies may be absent in up to 50% of patients with PNS, demonstrating their low sensitivity, which is in contrast to their relatively high specificity [4, 10]. 

Other laboratory tests of significance include tumour markers, which appeared to be the only investigative abnormality in one of the cases. The National Academy of Clinical Biochemistry does not favour requests for panels of tumour markers, recommending their use based on individualized clinical evaluations to yield the risk of malignancy [11, 12]. The specificity of Ca-125 is around 80%, indicating that a significant minority of patients may have elevated titres without an underlying ovarian neoplasm [13]. We could assume that, in the 2nd case, this was an erroneous result given the absence of a radiological abnormality. However, with hindsight, the elevated Ca-125 and the inflammatory changes observed at laparotomy could have been indications for radical oophorectomy. Diagnostic challenges in patients with PNS should be of no surprise therefore, given what may appear to be normal or conflicting results. 

Other authors have noted that a significant time interval may be present between the first presentation of, and discovery of, the underlying tumour, even after extensive investigations [14, 15]. Both Rees et al. and Patel et al. recommend the use of full-body positron emission tomography (PET) as the investigation of choice in such situations [16, 17]. The latter group found its sensitivity to be significantly greater than CT, though it is less specific [17]. Where PET is negative, current recommendations suggest patients should be screened initially after 3–6 months and 6 months thereafter, for a period of 4 years [18]. Thereafter, malignancy is unlikely [18]. Therefore, for the 2nd patient, a PET scan at an earlier stage could have provided further evidence for an underlying malignancy.

Corticosteroids, intravenous immunoglobulins, plasma exchange, and various other immunomodulatory therapies have been tried and can be effective in some paraneoplastic conditions such as NMDAR encephalitis [19]. However, to date, treatment of the underlying malignancy remains the best and most effective management for the paraneoplastic syndromes.

## 5. Conclusion

Patients presenting with features of a PNS should be investigated extensively for an underlying malignancy. After routine investigations, we recommend for patients to have tumour markers, paraneoplastic screen, imaging of the body including PET, and invasive procedures (i.e., biopsies and excision of “cysts”) if indicated. Ideally, the underlying malignancy should be managed prior to or in conjunction with the PNS.

## Figures and Tables

**Table 1:** Some of the known paraneoplastic syndromes with their associated antibodies and malignancies.

Syndrome	Antibodies	Associated malignancies
Cerebellar degeneration	Anti-Hu, Anti-Yo, Anti Ri, Anti CV2, and Anti-GAD, Anti-Tr, Anti-Zic4, Anti-mGluR1, and Anti-VGCC	SCLC, gynaecological, breast, thymoma, and others
Limbic encephalitis	NMDA, VGKC related antibodies, Anti-Hu, Anti-Ma, and Anti-GAD	Gynaecological, germ cell tumours of testis, and SCLC
Opsoclonus myoclonus	Anti- Ri, neuroleukin, gliadin, and Zic2	Gynaecological, breast, and SCLC
Brainstem encephalitis	Anti-Hu, Anti-Ri, and Anti-Ma	
Stiff person Syndrome	Antiamphiphysin, Anti-GAD	SCLC, breast,
Myelitis	Anti-Hu, Antiamphiphysin	
Peripheral neuropathy	Anti-CV2	SCLC, thymoma, and others
LEMS	Anti-VGCC	SCLC
